# Latent profile analysis of change fatigue and its relationship with work withdrawal behavior in clinical nurses: a multicenter study

**DOI:** 10.3389/fpubh.2025.1694873

**Published:** 2025-11-26

**Authors:** Xin Ma, Yi Li, Qiu-ping Ding, Ya-ping Zhang, Zhi-qin Bai, Yan-guang Su, Jia-cheng Shen, Jun-jun Huang, Si-yi Yang

**Affiliations:** 1 Department of Respiratory and Critical Care Medicine, Huzhou Central Hospital, Huzhou, Zhejiang, China; 2 Department of Encephalopathy, Kunshan Hospital of Integrated Chinese and Western Medicine, Kunshan, Jiangsu, China

**Keywords:** nurses, change fatigue, work withdrawal behavior, latent profile, influencing factors, relevance

## Abstract

**Objective:**

To investigate the latent categories of clinical nurses' change fatigue profiles, analyze the factors influencing these categories, and explore their correlation with work withdrawal behavior.

**Methods:**

A total of 733 nurses from various provinces and cities across China were selected as research subjects using convenience sampling. The subjects were investigated using a general information questionnaire, a change fatigue scale, and a work withdrawal behavior scale. The data were analyzed and tested through factor analysis and pairwise comparison.

**Results:**

Clinical nurses' change fatigue was categorized into three latent profiles: high, medium, and low change fatigue groups. Significant differences were observed in education level (χ^2^ = 20.968, *P* < 0.001), hospital level (χ^2^ = 12.021, *P* = 0.017), self-assessed work atmosphere (χ^2^ = 32.081, *P* < 0.001), self-assessed workload (χ^2^ = 44.677, *P* < 0.001), and coping style (χ^2^ = 13.346, *P* < 0.001) across the three groups of nurses. Logistic regression analysis indicated that education level, hospital level, self-assessed work atmosphere, workload, and coping style were influencing factors of change fatigue (*P* < 0.05). Nurses in the high change fatigue group exhibited significantly higher work withdrawal behavior than those in the medium group (*P* < 0.01), which was, in turn, significantly higher than in the low fatigue group (*P* < 0.01).

**Conclusions:**

For nurses experiencing change fatigue, which is closely linked to work withdrawal, managers should implement targeted interventions. These include optimizing the work environment, rational task allocation, and offering psychological support, all aimed at reducing withdrawal behaviors and promoting job stability.

## Introduction

Change fatigue describes the effect of rapid and continuous workplace changes on employees or managers, leading to intense stress, exhaustion, and burnout (1, 2). It is closely linked to organizational change. As nursing concepts evolve rapidly and the medical environment becomes increasingly complex, nurses are challenged to continually adapt to organizational shifts, such as changes in the environment, technology, and information. This constant adaptation is crucial for maintaining optimal nursing practices but also poses significant challenges for nurses and managers alike. Nurses in clinical settings must continuously learn to master new nursing knowledge and technical updates, changes in work processes, and the development of information systems to meet the evolving demands of their work (3, 4). This can negatively impact nurses' physical and mental health (5, 6).

The Conservation of Resources (COR) theory, proposed by psychologist Stevan E. Hobfoll in 1989, is a psychological theory that emphasizes the central role of resource dynamics in processes of stress and motivation. Its core assumption is that individuals have an innate motivation to acquire, retain, protect, and foster their valued resources. Stress reactions occur when individuals perceive a latent loss of these resources, an actual loss, or a failure to gain expected returns after investing resources. This theory has been widely applied in fields such as organizational behavior and human resource management, providing a robust framework for understanding phenomena like job burnout and work-family conflict (7).

Continuous organizational change can lead to change fatigue among nurses, which may jeopardize the successful implementation of organizational changes (8). Nurses may experience high mental stress, heavy workloads, emotional labor, and irregular work schedules, among other negative effects (9). Change fatigue has been identified as a critical barrier to work environment transformation (10). Studies indicate that the perpetually changing work environment and content in hospitals have become the norm for nurses, leading many to feel powerless, exhausted, disappointed, and passive about changes in their work content. This can even result in personality changes and increased work withdrawal behaviors, latently leading to resignation (11, 12). Work withdrawal behavior refers to negative responses to avoid work tasks when individuals are under pressure or dissatisfied with their work environment and role. It encompasses psychological withdrawal behaviors, such as avoidance and thoughts of leaving, as well as physical withdrawal behaviors, such as tardiness, early departure, procrastination, and absenteeism (13, 14). Nurses' work withdrawal behaviors can affect the quality of care, endanger patient safety, and ultimately lead to adverse outcomes for both individuals and the organization.

However, previous studies often treated nurses' change fatigue as a uniform phenomenon, neglecting latent group heterogeneity and individual differences. This has led to inconsistent research findings and difficulties in accurately evaluating and intervening for different groups of nurses experiencing change fatigue (4, 15). Therefore, analyzing the diverse profiles of nurses' change fatigue and developing prevention and intervention strategies for group change fatigue are crucial. Implementing timely and effective coping strategies can reduce work withdrawal behaviors and enhance career identity.

Current research into the correlation between change fatigue and work withdrawal behavior is primarily confined to cross-sectional examinations of contributing factors. This study utilizes latent profile analysis (LPA) to examine the latent profiles of change fatigue among clinical nurses, and to further investigate their characteristics and the correlation with work withdrawal behavior. The objective is to offer a reference for clinical medical staff to identify those with high levels of change fatigue.

## Study subjects and methods

### Study subjects

Nurses from 16 Class II Grade A and above hospitals in Hangzhou, Huzhou of Zhejiang Province; Nanjing, Xuzhou, Huai'an of Jiangsu Province; and Chongqing were selected as the research subjects using convenience sampling. Inclusion criteria: ① possession of a valid nurse practicing certificate and completion of the registration process; ② at least 6 months of clinical nursing experience; ③ voluntary participation in this study with a signed informed consent form. The exclusion criteria were: ① working in non-clinical positions; ② being temporarily absent from work during the investigation period due to sick leave or maternity leave. Based on a rough estimation, the sample size should be 15–20 times the maximum dimension of the survey scale, with a dropout rate of 15% to 20% taken into account. This study utilized 20 independent variables (12 items for general information + six items for the change fatigue scale + two dimensions for the work withdrawal behavior scale). The minimum sample size was calculated as *n* = 20 × 15 = 300. Considering a 15% sample dropout rate and invalid questionnaires, at least 353 cases were included to enhance statistical power. A total of 733 cases were included in this study. All participants volunteered for this study, which has been approved by the Ethics Committee of the Huzhou Central Hospital (Approval No. 2025030025).

### Survey tools

#### General information questionnaire

The general information questionnaire was compiled by the researchers. It included the nurses' general demographic information, the level of the hospital where they worked, their highest education level, their current professional title, monthly income, the number of night shifts (per month, the years of nursing work experience, the working environment atmosphere (rated as excellent, good, general, poor, or very poor), and their self-rated workload (categorized as much, more, normal, less, or very little). Additionally, it covered their coping style, which could be positive or negative.

#### Change Fatigue Scale (CFS)

Developed by Bernerth et al., a team of experts in human resource management from the United States, in 2011, the Change Fatigue Scale (CFS) is based on the theory of stress and organizational change. The scale was refined through several iterations (16), adhering to the Hinkin scale, and employs a Likert 7-point scoring method, ranging from “strongly disagree” to “strongly agree,” scored from 1 to 7 points. The total score spans from 6 to 42 points, with higher scores indicating greater fatigue among nurses in response to organizational change. The Chinese version of the scale has a Cronbach's α coefficient of 0.918, a split-half reliability of 0.911, and a test-retest reliability of 0.894. In this study, the Cronbach's α coefficient for the scale was 0.833.

#### Work Withdrawal Behavior Scale

The Work Withdrawal Behavior Scale was originally compiled by Lehman et al. (17) and adapted to Chinese by Yang Yazhong (18). This study references the revised version by Liu Jing (19), which consists of 12 items across two dimensions. It primarily assesses behaviors such as tardiness and resignation among nurses. A Likert 5-point scale is used, with 1 indicating “never” and 5 indicating “always.” The total score ranges from 12 to 60, where higher scores suggest more frequent work withdrawal behavior. The scale's Cronbach's α coefficient is 0.90, and in this study, it was 0.824.

### Data collection methods

This study adheres to the principle of voluntariness. Clear instructions are provided on the homepage and each section of the questionnaire. The same IP address is restricted to a single submission at any given time. The questionnaire's QR code was disseminated by the researcher via the director of the nursing department, head nurse, and clinical nurses. The survey was conducted anonymously, with no personal information disclosed or personal factors discussed separately. The test-retest reliability method was employed to assess the questionnaire's consistency. Three questions with identical stems but different options were positioned in various parts of the questionnaire. Questionnaires with varying options and response times of < 5 min were deemed invalid to ensure the questionnaire's reliability and to confirm that each accurately reflects the true sentiments of clinical nurses. To maintain the enthusiasm of new nurses, WeChat red envelopes valued at 5–10 yuan were offered. Initially, 733 questionnaires were distributed, and after excluding 12 invalid ones, the effective recovery rate was 98.91%.

### Statistical methods

Count data were described using frequency and constituent ratios, while measurement data that conformed to a normal distribution were presented as mean ± standard deviation. Measurement data that did not meet the criteria for normal distribution were expressed as M (P25, P75). The *t*-test and chi-square test were employed to analyze the influencing factors between groups to assess the impact of various factors on different latent profiles. Mplus 8.3 software was utilized to analyze the latent profile categories of clinical nurses' change fatigue. Initially, the data model C1 was constructed, and the number of categories was incrementally increased. The optimal fitting model was selected by comparing the log-likelihood function value (Log(L)), Akaike information criterion (AIC), Bayesian information criterion (BIC), and the sample-size adjusted BIC (aBIC) values. Furthermore, the Lo-Mendell-Rubin adjusted likelihood ratio test (LRT), bootstrap-based likelihood ratio test (BLRT), and *P*-value were commonly used to evaluate the model fit. The entropy value was used to assess the accuracy of classification, with values closer to 1 indicating higher classification accuracy.

## Results

### Basic information and change fatigue scale scores

The information pertaining to 725 nurses is presented in Table 1. The scores for change fatigue ranged from 8 to 42, with a total score of 24.33 ± 5.71 and an average item score of 4.06 ± 0.75.

**Table 1:** General information of respondents (*n* = 725).

**Items**	**Categories**	***n***	**Percentage (%)**	**Items**	**Categories**	***n***	**Percentage (%)**
Age (years)	< 30	197	27.17	Number of night shifts (months)	< 3	131	18.07
	30–45	304	41.93		3–6	387	53.38
	>45	224	30.90		>6	207	28.55
Gender	Male	35	4.83	Length of service (years)	< 5	190	26.21
	Female	690	95.17		5–9	265	36.55
Level of education	College or below	161	22.21		10–19	134	18.48
	Undergraduate	514	70.90		≥20	136	18.76
	Master's degree or above	50	6.89	Self-rated work atmosphere	good	81	11.17
Monthly income (yuan)	< 4,000	122	16.83		Better	218	30.07
	4,000–	222	30.62		General	295	40.69
	7,000–	274	37.79		Poor	92	12.69
	>10,000	107	14.76		poor	39	5.38
Marital status	Unmarried	153	21.10	Self-rated workload	more	237	32.69
	Married	551	76.00		More	296	40.83
	Divorce	21	2.90		Normal	160	22.07
Hospital grade	Class II Grade A	518	71.45		Less	26	3.59
	3rd Grade B	133	18.34		less	6	0.82
	3rd Grade A	74	10.21	Coping style	Coping positively	472	65.10
Current title	Junior professional title	238	32.83		Negative coping	253	34.90
	Intermediate title	343	47.31				
	Senior title	144	19.86				

### Latent profile analysis of change fatigue in clinical nurses

To determine the latent profile of change fatigue among clinical nurses, researchers selected the scores of six items as assessment indicators for the score of change fatigue. They began with a baseline model (with the number of categories set to one) and successively fitted and analyzed models containing one to four latent profiles. The specific data are presented in Table 2. After thorough consideration and evaluation, three latent profile models were ultimately identified as the optimal models for clinical nurses' change fatigue.

**Table 2:** Comparison of fitting parameter indexes for various latent profile models (*n* = 725).

**Models**	**AIC**	**BIC**	**aBIC**	**Entropy**	**LRT**	**BLRT**	**Class probability**
1	17,185.471	17,240.489	17,202.385				
2	16,693.362	16,780.473	16,720.143	0.823	0.000	< 0.001	0.37/0.63
3	16,447.806	16,567.046	16,484.488	0.845	0.010	< 0.001	0.25/0.28/0.47
4	16,288.521	16,439.820	16,335.035	0.815	0.134	< 0.001	0.29/0.15/0.23/0.33

### The average attribution rate of three latent categories of change fatigue among clinical nurses

To verify the reliability of the three latent profile category analysis results, the average attribution probability for each of the three class samples was calculated. The results indicate that the average attribution probability ranges from 87.5 to 94.4%, all exceeding 90%, suggesting that the three latent profiles are reliable, as illustrated in Table 3.

**Table 3:** Average attribution probability of three types of change fatigue (*n* = 725).

**Model**	**C1**	**C2**	**C3**
C1	0.890	0.110	0.000
C2	0.021	0.944	0.035
C3	0.000	0.125	0.875

### Latent category characteristics of change fatigue in clinical nurses

**Figure 1:**
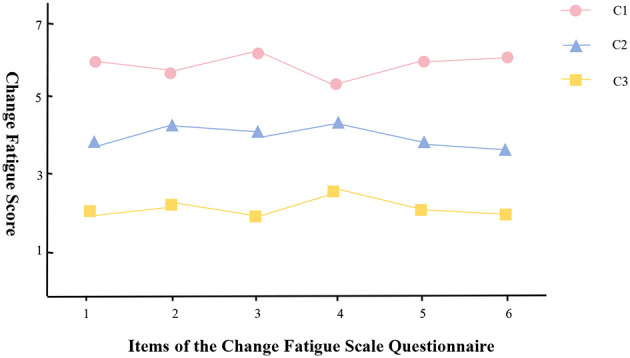
1: The organization has introduced too many change measures. 2: I'm tired of all the changes in the organization. 3: In an organization, the number of changes taking place is overwhelming. 4: In the organization, we are required to change too many things. 5: It feels like we are always required to make all kinds of changes. 6: Before the organization implements any changes, I hope there can be a stable period.

To clearly and precisely define the changing trends of different sections of clinical nurses' change fatigue, a line graph of the scores of each dimension of the change fatigue scale was created (see Figure 1). This enables us to more clearly analyze the characteristics of change fatigue among these three latent categories of clinical nurses, we can more clearly delineate the characteristics of change fatigue among clinical nurses within these three latent categories. The three categories have been designated as follows: Category 1 (C1), referred to as the “high change fatigue group,” comprises 28.14% of the cases (204 nurses); Category 2 (C2), known as the “medium change fatigue group,” includes 46.90% (340 nurses); and Category 3 (C3), termed the “low change fatigue group,” encompasses 24.96% (181 nurses).

### Single factor analysis of latent categories of clinical nurses' change fatigue

To clarify the influencing factors of different profiles of clinical nurses' change fatigue, a univariate analysis was first conducted. Univariate analysis revealed significant differences in education level (χ^2^ = 20.968, *P* < 0.001), hospital level (χ^2^ = 12.021, *P* = 0.017), self-rated work climate (χ^2^ = 32.081, *P* < 0.001), and self-rated workload (χ^2^ = 44.677, *P* < 0.001) among the three categories of nurses. Coping style also showed significant differences (χ^2^ = 13.346, *P* < 0.001), as indicated in Table 4.

**Table 4:** Single-factor analysis of general information and latent profiles of change fatigue (*n* = 725).

**Variables**	**Group C1 (*n* = 204)**	**Group C2 (*n* = 340)**	**Group C3 (*n* = 181)**	**χ^2^**	***P*-value**
**Age (years)**				8.674	0.070
< 30	54	103	40		
30–45	83	148	73		
>45	67	89	68		
**Gender**				0.218	0.897
Male	11	16	8		
Female	193	324	173		
**Level of education**				20.968	< 0.001
Junior college and below	64	55	42		
Undergraduate	130	263	121		
Master's degree or above	10	22	18		
**Monthly income (Yuan)**				6.579	0.362
< 4,000	37	59	26		
4,000–	64	91	67		
7,000–	75	135	64		
>10,000	28	55	24		
**Marital status**				2.962	0.564
Unmarried	45	77	31		
Married	153	252	146		
Divorced	6	11	4		
**Hospital grade**				12.021	0.017
Class II Grade A	151	254	113		
3rd Grade B	34	51	48		
3rd Class A	19	35	20		
**Current title**				7.777	0.100
Junior title	82	101	55		
Intermediate title	88	170	85		
Senior title	34	69	41		
**Number of night shifts (months)**				3.812	0.432
< 3	33	58	40		
3–6	106	188	93		
>6	65	94	48		
**Length of service (years)**				9.674	0.139
< 5	61	96	33		
5–9	72	124	69		
10–19	32	61	41		
≥20	39	59	38		
**Self-rated work climate**				32.081	< 0.001
Good	10	38	33		
Better	64	89	65		
Average	95	136	64		
Poor	24	55	13		
Poor	11	22	6		
**Self-rated workload**				44.677	< 0.001
More	83	115	39		
More	95	131	70		
Normal	18	78	64		
Less	7	13	6		
less	1	3	2		
**Style of coping**				17.295	< 0.001
Coping positively	112	225	135		
Negative coping	92	116	45		

“C1 represents the “high change fatigue group,” C2 represents the “medium change fatigue group,” and C3 represents the “low change fatigue group.”

### Logistic regression analysis of latent categories of change fatigue in clinical nurses

To clarify the influencing factors of different latent profiles of change fatigue among clinical nurses, Logistic regression analysis is required. Logistic regression analysis was conducted with the clinical nurses' change fatigue group as the dependent variable and the indicators that showed statistically significant differences in univariate analysis as the independent variables. The results indicated that, in comparison with group C3, the level of education (with the assignment: Specialized subject and below = 1, undergraduate course = 2, master's and above = 3, using specialized subject and below as the control), hospital grade (level 2 grade A = 1, 3rd Grade B = 2, 3rd Class A = 3, with level 2 grade A as the control), and the appraisal of the work atmosphere (good = 1, better = 2, generally = 3, poor = 4, with good as the contrast), self-reported workload (most = 1, more = 2, normal = 3, less = 4, with most as the control), and coping style (positive coping = 1, negative coping = 2, using positive coping as the control) were significant predictors. Logistic regression analysis revealed that the level of education, hospital grade, self-rated work atmosphere, workload, and coping style were influencing factors of change fatigue (*P* < 0.05). The data is presented in Table 5.

**Table 5:** Logistic regression analysis of the change trajectory of clinical nurses' change fatigue (*n* = 725).

**Items**	***B*-value**	**SD**	**Walds χ^2^**	***P*-value**	**OR**	**Upper limit of 95% CI**	**Lower limit of 95% CI**
**Group C1 vs. group C3**
Constants	2.133	0.314	46.145	< 0.001			
**Level of education**
Undergraduate	−0.265	0.140	3.583	0.060	0.767	0.583	1.009
Master's degree or above	−0.356	0.094	14.343	< 0.001	0.700	0.583	0.842
**Hospital grade**
Level 3 Grade B	0.288	0.067	18.477	< 0.001	1.334	1.170	1.521
3rd Grade A	0.564	0.163	11.972	< 0.001	1.758	1.277	2.419
**Self-rated work climate**
Better	0.216	0.122	3.135	0.066	1.241	0.977	1.576
Average	0.333	0.164	3.663	0.059	1.395	0.992	1.962
Poor	0.417	0.134	9.684	0.001	1.517	1.167	1.973
Poor	0.489	0.155	9.953	< 0.001	1.631	1.203	2.210
**Self-rated workload**
More	−0.303	0.156	3.773	0.057	0.739	0.544	1.003
Average	−0.386	0.198	3.801	0.056	0.680	0.461	1.002
Less	−0.406	0.141	8.291	0.008	0.666	0.505	0.878
less	−0.457	0.139	10.809	< 0.001	0.633	0.482	0.831
**Coping style**
Negative coping	0.477	0.195	5.984	0.021	1.611	1.099	2.361
**Group C2 vs. group C3**
Constants	1.965	0.249	62.277	< 0.001			
**Level of education**
Undergraduate	−0.402	0.137	8.610	0.006	0.669	0.511	0.875
Master's degree or above	−0.466	0.203	5.270	0.030	0.628	0.422	0.934
**Grade of hospital**
Level 3 Grade B	0.305	0.082	13.835	< 0.001	1.357	1.155	1.593
3rd Grade A	0.439	0.130	11.404	< 0.001	1.551	1.202	2.001
**Self-rated work climate**
Better	−0.196	0.125	2.459	0.123	0.822	0.643	1.050
Average	−0.207	0.143	2.095	0.157	0.813	0.614	1.076
Poor	0.238	0.095	6.276	0.018	1.269	1.053	1.528
poor	0.303	0.088	11.856	< 0.001	1.354	1.139	1.609
**Self-rated workload**
More	−0.175	0.102	2.944	0.073	0.839	0.687	1.025
Average	−0.224	0.139	2.597	0.120	0.799	0.609	1.050
Less	−0.311	0.118	6.946	0.013	0.733	0.581	0.923
less	0.343	0.104	10.877	< 0.001	1.409	1.149	1.728
**Coping style**
Negative coping	0.415	0.108	14.766	< 0.001	1.514	1.225	1.871

“C1 represents the “high change fatigue group,” C2 represents the “moderate change fatigue group,” and C3 represents the “low change fatigue group.”

### Differences in work withdrawal behavior of clinical nurses with different profiles of change fatigue

The work withdrawal behavior score of 725 clinical nurses was (37.33 ± 8.76). To understand the differences in job withdrawal behaviors of nurses with change fatigue in different profiles, the scores of job withdrawal behaviors of clinical nurses in different change fatigue profiles were analyzed and compared. The findings indicated that the work withdrawal behavior level of nurses in the high change fatigue group was significantly higher than that of the medium change fatigue group (*P* < 0.01) and the low change fatigue group (*P* < 0.01). Additionally, the work withdrawal behavior level of nurses in the medium change fatigue group was significantly higher than that of the low change fatigue group (*P* < 0.01), as presented in Table 6.

**Table 6:** Differences in work withdrawal behavior among clinical nurses with various change fatigue profiles.

**Variables**	**Group C1**	**Group C2**	**Group C3**	***F***	**Compare the results in pairs**
Psychological withdrawal behavior dimension	20.45 ± 3.11	18.39 ± 3.56	15.78 ± 2.43	9.256	C1 > C3^**^, C1 > C2^**^, C2 > C3
Physical withdrawal behavior dimension	19.44 ± 2.95	18.95 ± 3.04	18.65 ± 3.60	3.923	C1 > C3^**^, C1 > C2
Total score on work withdrawal behavior	39.89 ± 6.67	37.34 ± 6.44	34.43 ± 6.57	13.705	C1 > C3^**^, C1 > C2^**^, C2 > C3

“C1 represents the “high change fatigue group,” C2 represents the “medium change fatigue group,” and C3 represents the “low change fatigue group.” An asterisk (^*^) indicates *P* < 0.05, while a double asterisk (^**^) signifies *P* < 0.01.

## Discussion

### Clinical nurses' change fatigue is at a medium level

The results of this study indicate that clinical nurses experience a moderate level of change fatigue. This is primarily attributed to the professional and operational characteristics of nursing work and the medical system, respectively. The professional resilience developed through nursing education can effectively mitigate the pressures associated with certain changes. Despite the continuous updates in medical policies and technologies, the core duties of nursing have largely remained constant, as supported by Fernemark et al.'s research (20). The moderate level of fatigue signifies an adaptive equilibrium among nurses during the change process, which not only preserves the requisite occupational alertness but also guarantees fundamental work efficiency. This condition reflects not only the professional caliber of the nursing staff but also suggests that medical institutions might consider a gradual approach, allowing nursing personnel a suitable period for adaptation when implementing reforms.

### Clinical nurses' change fatigue had three different profile categories

This study, utilizing Latent Profile Analysis (LPA), identified three latent profile categories of clinical nurses' change fatigue: high, medium, and low. This finding underscores the heterogeneity in the change fatigue status among clinical nurses. In the study, change fatigue was examined from an internal perspective, and clinical nurses were categorized based on their levels of change fatigue, facilitating the differentiation of various groups for targeted interventions. The high change fatigue group (comprising 28.14% of the nurses, exhibited significant challenges in adapting to changes). This difficulty may be associated with prolonged exposure to high-intensity work environments or frequent systemic reforms (21). Nurses in this group tend to have negative perceptions of organizational changes and are susceptible to emotional exhaustion and job burnout, which can lead to reduced work efficiency. Their elevated fatigue may stem from insufficient personal resources, such as a lack of social support or weak psychological resilience (22, 23). Management should concentrate on this group, implementing stress-reduction interventions and personalized support to assist them in regaining confidence in change. The moderate change fatigue group, at 46.90%, was the largest, with an intermediate level of change fatigue that is manageable. These nurses generally possess basic adaptive capabilities but may experience periodic pressure when faced with additional changes. The organization can maintain the stability of this group by enhancing the change communication mechanism and offering transitional training to prevent them from transitioning into the high fatigue group. The dynamics of this group also reflect a common adaptive threshold for the intensity of change within the current healthcare system. Nurses in the low change fatigue group, constituting 24.96%, demonstrated robust adaptability to change. They typically employ positive coping strategies and possess sufficient psychological resources. Their characteristics may include a strong professional identity, flexible problem-solving skills, or a supportive organizational environment. These nurses tend to view change as an opportunity rather than a threat, and their lower levels of fatigue contribute to maintaining team stability.

### Analysis of influencing factors of three different profile categories of clinical nurses' change fatigue

The results of this study indicated significant differences in clinical nurses‘ susceptibility to change fatigue, which were associated with their education level, hospital level, self-assessed work atmosphere, workload, and coping style. Willis et al. noted that nurses with higher education typically possess more systematic professional knowledge and stronger learning adaptability, enabling them to better understand and cope with the challenges posed by medical reforms, thus experiencing relatively lower levels of change fatigue (24). The influence of hospital level is primarily due to the fact that higher-level hospitals are at the forefront of implementing intensive reforms, necessitating nurses to continuously adapt to new technologies and processes. Additionally, nurses are expected to manage heavier diagnostic and treatment tasks and adhere to stricter quality standards, with the pressure of reform compounding the already high-pressure work environment. Furthermore, the stringent assessment systems directly convert the costs of adaptation to reform into personal burdens. Furthermore, Garside et al. pointed out that nurses need to devote more energy to adapting to changes in order to maintain their professional advantages, and the complex management hierarchy may also delay the implementation of support measures, jointly exacerbating fatigue (25). Departments with a positive self-assessed work atmosphere generally have more open communication channels and more harmonious colleague relationships. This supportive environment can effectively mitigate the pressures of change, whereas a negative atmosphere may exacerbate feelings of isolation and helplessness among nurses. An excessive workload directly depletes nurses' physical and mental resources, diminishing their capacity to manage additional changes. Particularly, when changes impose extra workloads, the resulting fatigue is significantly heightened (26). Coping style is the most influential individual factor. Nurses who adopt proactive coping strategies, such as active learning and seeking support, are better equipped to adapt to change, while those who resort to passive avoidance are more prone to accumulating fatigue (10). The interplay of these factors collectively shapes the varied responses of nurses to medical system reforms, offering a crucial foundation for targeted interventions.

### Correlation between different change fatigue ability and work withdrawal behavior of clinical nurses and its guiding effect on clinical practice

This study revealed that the overall level of work withdrawal behavior among clinical nurses was moderate. Specifically, work withdrawal behavior demonstrated a clear gradient relationship with the severity of change fatigue, meaning that groups with higher levels of fatigue exhibited significantly higher levels of this behavior. These findings suggest that change fatigue is a critical stressor influencing nurses' professional conduct (27). When clinical nurses are subjected to a high-intensity, long-term change environment without adequate adaptation, they may experience emotional exhaustion and cognitive overload, subsequently seeking relief through psychological detachment and behavioral avoidance (28, 29). This implies that clinical managers should establish a dynamic monitoring system for change fatigue, conduct targeted interventions for those with high levels of fatigue, streamline work processes to minimize the burden of unnecessary changes, offer psychological counseling and team support to bolster coping mechanisms, and ensure nurses have a say in changes through participatory management. Additionally, it is essential to enhance the hospital's change management system, providing appropriate resource support and adaptation training during the implementation of reforms to mitigate the impact on the nursing team and maintain work stability.

## Limitations

This study has certain limitations. Sampling was only conducted in China, which may lead to a decrease in the universality level of the sample. In addition, adopting the form of self-reporting outcomes may increase the risk of reporting bias. Subsequently, the research results will be optimized in response to the above issues.

## Summary

In this study, latent profile analysis was utilized to categorize clinical nurses' change fatigue into three distinct latent profile groups: high, medium, and low. These three groups were influenced by factors such as education level, hospital tier, self-assessed work environment, workload, and coping mechanisms. The varying profiles of change fatigue are closely associated with the extent of work withdrawal behavior exhibited by nurses. Future research should further investigate the specific formation mechanisms underlying different change fatigue profiles. For instance, it is essential to explore whether nurses in the high change fatigue group exhibit specific risk factors related to emotional labor, work-family conflict, or social support, and to longitudinally track the evolution trajectories of these profiles. At the application level, targeted intervention plans can be developed based on profile characteristics. This includes providing intensified psychological resilience training and multidisciplinary support for nurses in the high fatigue group, while optimizing work processes and change management strategies for those in the medium-to-low fatigue groups. Furthermore, integrating this profiling system into the nurse health management system would facilitate early identification and stratified management.

## Data Availability

The original contributions presented in the study are included in the article/supplementary material, further inquiries can be directed to the corresponding author.
